# Application of Bis-Adducts of Phenyl-C_61_ Butyric Acid Methyl Ester in Promoting the Open-Circuit Voltage of Indoor Organic Photovoltaics

**DOI:** 10.3390/ma16072613

**Published:** 2023-03-25

**Authors:** Xueyan Hou, Xiaohan Duan, Mengnan Liang, Zixuan Wang, Dong Yan

**Affiliations:** 1 International Collaborative Laboratory of 2D Materials for Optoelectronics Science and Technology of Ministry of Education, Institute of Microscale Optoelectronics, Shenzhen University, Shenzhen 518060, China; 2 Guangdong-Hong Kong-Macao Joint Laboratory for Intelligent Micro-Nano Optoelectronic Technology, School of Physics and Optoelectronic Engineering, Foshan University, Foshan 528225, China; 3 School of Mechanical Engineering and Automation, Northeastern University, Shenyang 110819, China

**Keywords:** indoor photovoltaics, fullerene derivatives, open-circuit voltage, internet of things

## Abstract

Fullerene-based indoor OPVs, particularly phenyl-C_61_ butyric acid methyl ester (PCBM), has been regarded as a prospective harvesting indoor light energy source to drive low-power consumption electronic devices such as sensors and IoTs. Due to the low tunability of its inherently spherical structure, the performance of the fullerene-based indoor OPVs seem to hit a bottleneck compared with the non-fullerene materials. Here, we explore the potential application of fullerene derivative bis-PCBM in indoor OPVs, which owns a higher the lowest unoccupied molecular orbital (LUMO) level than PCBM. The results show that when blended with PCDTBT, bis-PCBM devices yield a high *V*_OC_ of up to 1.05 V and 0.9 V under AM 1.5G illumination and 1000 lx indoor light, compared with the corresponding values of 0.93 V and 0.79 V for PCBM devices. Nevertheless, the disorders in bis-PCBM suppress the *J*_SC_ and FF and, therefore, result in a lower efficiency compared to PCBM devices. However, the efficiency and stability differences between the two kinds of cells were much reduced under indoor light conditions. After further optimization of the material composition and fabrication process, bis-PCBM could be an alternative to PCBM, offering great potential for indoor OPV with high performance.

## 1. Introduction

Organic photovoltaics (OPVs) are considered to be a highly promising candidate for a large-area, flexible, and low-cost renewable energy source. Even though considerable progress has been made in the power conversion efficiencies (PCE) of OPVs; however, stability issues under harsh outdoor environments are a drawback for the commercialization of these devices [1,2,3]. The indoor environment has very different environmental stress factors associated with low light conditions, e.g., lack of elevated temperatures, intensive light socking, thermal cycling, and weathering, which can provide an alternative scenario for OPV commercialization. In the past few years, indoor PVs have attracted intense research attention due to their potential to harvest indoor light energy to drive low-power consumption electronic devices, such as indoor sensors and the internet of things (IoT) [4]. Among them, OPV-based indoor PVs have emerged as promising candidates for efficient light-harvesting technologies when employing highly efficient fullerene and non-fullerene materials as acceptors [5,6,7,8]. The highest efficiency of indoor OPVs based on fullerene materials is 28% under 1000 illuminance (lx) indoor light [6], and the optimized indoor OPVs based on non-fullerene materials exhibited a record PCE up to 33.2% under 19,500 lx LED light [9]. Due to the diverse structure of non-fullerene materials, as well as their remarkable optoelectronic characteristics, such as excellent light-harvesting ability and adjustable energy level, most of the current indoor OPV research is based on non-fullerene materials [5,9,10,11]. In contrast, fullerene materials have relatively low tunability due to their inherently spherical structure; thus, it seems that there is not much room for performance improvement for the fullerene-based, particularly PCBM, indoor OPVs.

However, fullerene materials could have greater application prospects than expected. PCBM and PC_71_BM have been utilized in indoor photovoltaic materials and achieved high efficiency [6,12,13]. Fullerene materials are usually adopted in ternary OPV devices, which include a third component in the original binary OPV devices [14]. This strategy can overcome the original devices’ light absorption limitations and enhance the active layer’s morphology and energy level alignments, thus increasing the PCE of OPVs [14]. The blue light emitted by indoor PVs can impair vision, and studies have shown that adding fullerene can lessen the effect [15]. Furthermore, commercial fullerene materials are cheaper than non-fullerene materials. Studies have shown that open-circuit voltage (*V*_OC_) is important for the operation of indoor OPVs [7]. Fullerene derivatives, such as bis-PCBM, can increase the lowest unoccupied molecular orbital (LUMO), thereby increasing *V*_OC_ [16,17]. To our best knowledge, indoor OPVs based on bis-PCBM have not yet been studied. Thus, exploring the potential application of the fullerene derivative bis-PCBM in indoor OPVs is essential, as they own higher LUMO levels but have more disorders than PCBM.

**Figure 1:**
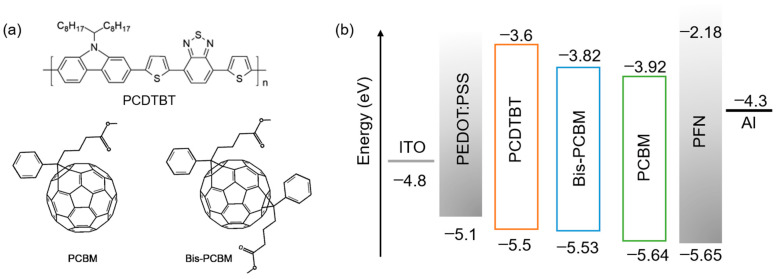
The chemical structure (**a**) and energy band diagram (**b**) of the polymer PCDTBT and fullerene acceptor PCBM and bis-PCBM. The chemical structure of bis-PCBM is a general one without considering its multi-isomers with the second side chain at different positions.

The work in this paper employed bis-PCBM and the counterpart PCBM with PCDTBT in indoor OPV devices for the first time. The molecular structures and corresponding energy levels are shown in Figure 1 (the energies are from references [18,19,20]). The device performance indicated the positive effects of bis-PCBM on the *V*_OC_ under both AM 1.5G and indoor light. Although the PCE of bis-PCBM devices did not surpass that of the PCBM devices due to disorders and the amorphous property, the PCE and stability differences between the two kinds of devices were much reduced under indoor low light conditions. Bis-PCBM is expected to be a promising replacement for PCBM in high-efficient indoor OPVs with elevated *V*_OC_ and enhanced PCE via material synthesis and fabrication process optimization.

## 2. Materials and Methods

Materials: The regents and solvents were used as received from Sigma Aldrich (St. Louis, MI, USA). The materials were purchased from 1-materials.

**Figure 2:**
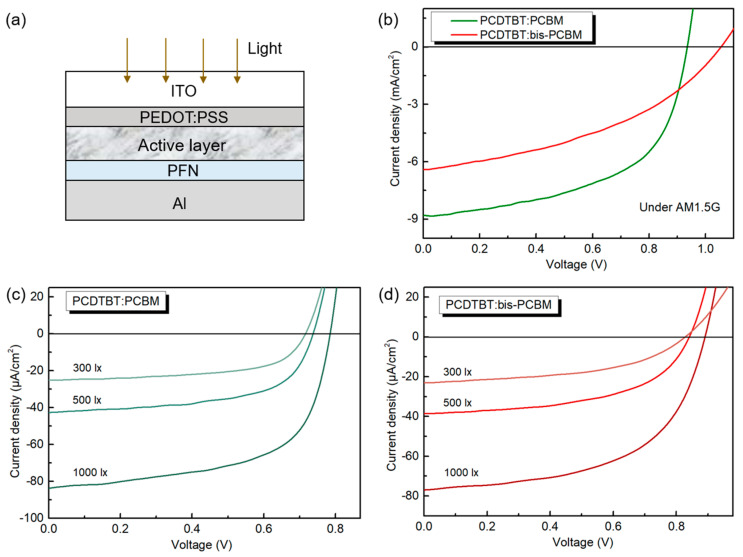
(**a**) Schematic diagram of the device structure. (**b**–**d**) *J*-*V* characteristics of the PCDTBT:PCBM and PCDTBT:bis-PCBM devices under AM 1.5G and indoor illuminations with the light intensity of 300 lx, 500 lx, and 1000 lx.

Device fabrication and characterization: The schematic structure of the device used in this study is shown in Figure 2a. All layers were fabricated by spin coating from corresponding solutions. The metal electrode was thermally evaporated. The ITO glass (~1.1 mm) with an ITO thickness of ~100 nm and sheet resistance of ~15 Ω per square was prepared after cleaning and UV ozone treatment. The PEDOT:PSS electron transport layer was spin-coated on the ITO with a thickness of ~30 nm, followed by annealing at 150 °C for 20 min. The PCDTBT:fullerene blend solutions were prepared by dissolving solid PCDTBT and fullerene derivatives in chlorobenzene with a weight ratio of 1:2 and concentration of 20 mg/mL. The active layer was spun onto the ITO/PEDOT:PSS substrates with a thickness of ~85 nm under a nitrogen atmosphere. The film thicknesses were measured with a Dektak XT profilometer (Bruker, MA, USA). After the film was dry, the active layers were carried out with the solvent vapor annealing (SVA) treatment in a glass petri dish containing a THF atmosphere. The PFN was dissolved in methanol + 0.25 vol% acetic acid with a concentration of 0.2 mg/mL. Then the PFN was spin-cast on the active layer with a thickness of ~5 nm. Subsequently, the films were transferred to an evaporator for Al cathode deposition (100 nm). During the thermal evaporation of the Al electrode, a 6-pixel mask was used on the top of the substrate with a well-defined pixel area of 0.045 cm^2^. The current density–voltage (*J*-*V*) characterization was carried out using a Keithley 2400 Source (Tektronix, OR, USA) measurement unit under the solar simulator (AM 1.5G standard light) or fluorescent lamps. The lux level was measured using a Luxmeter (Fluke, DC, USA). The EQE spectra were obtained using a grating spectrometer to create monochromatic light from a tungsten halogen light source in combination with a series of filters and a Stanford Research System SR380 lock-in amplifier to detect the photocurrent. A Silicon photodiode was used to calibrate the spectra.

Degradation: Only the active layer coated ITO/PEDOT:PSS structures were used to perform the degradation treatment under AM 1.5G irradiation or 300 lx fluorescent light in air for different periods of time. After that, the interlayer and top electrode were fabricated to finish the devices.

## 3. Results and Discussion

### 3.1. Device Performance

OPV devices based on the conventional structure of ITO/PEDOT:PSS/active layer/PFN/Al (as shown in Figure 2a) were fabricated to investigate the effect of different cases of electron acceptors on the photovoltaic performance under both AM 1.5G and indoor lighting conditions. Figure 2b–d presents the experimentally measured typical *J*-*V* curves. The detailed device parameters are collected in Table 1. Under AM 1.5G light, PCDTBT:bis-PCBM cells exhibited a high *V*_OC_ of up to 1.05 eV, which was 120 meV higher than the *V*_OC_ value of the PCDTBT:PCBM devices (0.93 eV) due to the high-lying LUMO level of bis-PCBM. Nevertheless, the PCE of the PCDTBT:bis-PCBM cells (~2.9%) was much lower than that of the PCDTBT:PCBM cells (~4.7%). Of the performance parameters, the fill factor (FF) and short circuit current density (*J*_SC_) were the short boards in the PCDTBT:bis-PCBM-based OPV devices, which were only 0.43 and 6.4 mA/cm^2^, respectively (0.59 and 8.5 mA/cm^2^ for PCDTBT:PCBM-based device). Earlier studies found that when using fullerene, higher adducts did not result in improved PCE due to a number of reasons: the addition of the second side chain on bis-PCBM inhibits the close packing of C_60_ cage; bis-PCBM is amorphous; bis-PCBM is a mixture of multiple isomers which introduce disorders into both the electronic energy levels and molecular packing resulting in an adverse effect on electronic properties [21]; and the driving force for exciton dissociation at the PCDTBT/bis-PCBM interface is smaller than that of the PCDTBT/PCBM interface. These factors lead to insufficient charge transport (or electron mobility), charge injection and extraction, charge separation, and morphology problems [22,23].

The light intensities under indoor environments are usually around 100–1000 lx [24]. Therefore, we studied the photovoltaic performance of the bis-PCBM and PCBM-based OPV cells under three light intensities in this range (e.g., 300 lx, 500 lx, and 1000 lx). As the light intensity increases, all the OPV cells show significantly increased *J*_SC_ and *V*_OC_ due to the increased input photon number. The FFs also increased slightly, leading to the overall increase in PCE from 13.2 to 14.7% (PCBM cells) and from 12 to 13.8% (bis-PCBM cells). Under the same light intensity, the *J*_SC_ values of bis-PCBM and PCBM-based OPV devices did not show as many differences as when under AM 1.5G illumination, which was around 25, 40, and 80 μA/cm^2^ under 300, 500, and 1000 lx intensity, respectively. This may be due to the fact that all fullerene devices can effectively utilize indoor light. In comparison with the results obtained under AM 1.5G, the bis-PCBM-based devices also showed a higher *V*_OC_ value (110 meV) than the PCBM-based devices, while the FFs were still very low in the range of 0.52–0.55 compared to the FF range of PCBM (0.61–0.62). Overall, the disadvantage in FF of the bis-PCBM cells offset their advantage in *V*_OC_, leading to an overall lower PCE than seen in PCBM cells. The performance of bis-PCBM-based indoor PVs is still promising compared with other polymer:fullerene systems, such as the P3HT:PCBM, PBDB-T:PCBM, and PTB7:PCBM blends, which exhibited similar PCEs in the range of 9 to 15% but much lower *V*_OC_ (0.5 V, 0.67 V, and 0.57 V, respectively) under 1000 lx indoor light [25]. Replacing PCBM in these polymer:fullerene systems with bis-PCBM would improve the *V*_OC_ and potentially enhance the device performance in an indoor environment. Our work demonstrates that bis-PCBM has a higher indoor PV application prospect than mono-fullerene PCBM.

### 3.2. Device Degradation

**Figure 3:**
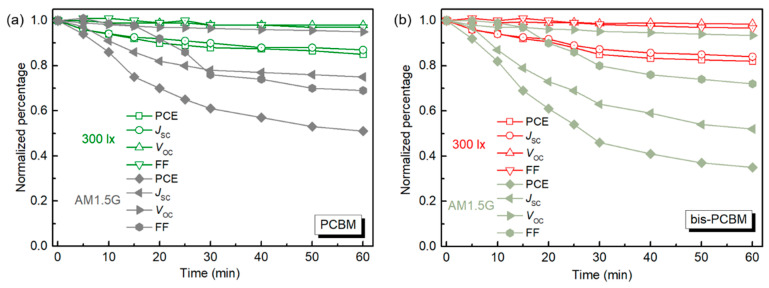
The normalized device performance parameters of the OPV devices based on PCDTBT blended with PCBM (**a**) and bis-PCBM (**b**) after degradation under AM 1.5G or 300 lx indoor light, in air, for a different time. All performance parameters are normalized to the value measured for the undegraded device.

Following the device performance study, we proceeded to investigate the degradation of the bis-PCBM and PCBM-based OPV devices after exposure of the active layer to AM 1.5G illumination or 300 lx indoor light, in air, for different periods. The degradation was carried out before the deposition of the top interlayer and electrode. The evolution of device performance parameters as a function of exposure time to light is summarized in Figure 3. All values were normalized to their respective initial values before degradation. It is obvious that the bis-PCBM and PCBM device parameters exhibited a range of degradation trends and the devices showed a slower degradation rate under low light intensity than that under AM 1.5G illumination. Under AM 1.5G, only the *V*_OC_ does not change much, maintaining >93% of the original value. The other parameters, *J*_SC_, FF, and PCE, dropped faster in the initial 20 min and then leveled off until 60 min. The PCE remains 51% for PCBM and 35% for bis-PCBM after 60 min of degradation. This difference in degradation performance of bis-PCBM and PCBM-based blends may be due to crystalline PCBM having a lower tendency to take part in the oxidation reaction due to the denser molecular packing inhibiting the permeation of oxygen. Furthermore, the PCDTBT:fullerene blend degrades through a superoxide (O_2_^−^) degradation mechanism, whereby a higher LUMO of bis-PCBM speeds up the rate of electron transfer from fullerene to oxygen to form O_2_^–^ [26]. Under irradiation at 300 lx, PCE of both PCDTBT:fullerene devices maintain over 80% of the original value after 60 min (85% for PCBM, 82% for bis-PCBM). Among the device parameters, the *V*_OC_ and FF of the two kinds of devices maintain very high stability under 300 lx and keep >97% of the initial values after continuous irradiation for 60 min. It is the reduced *J*_SC_ that accounts for the main PCE loss of the devices under low light.

**Figure 4:**
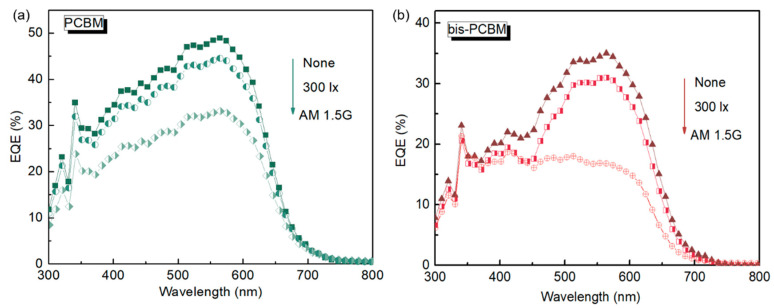
EQE of the OPV devices based on PCDTBT blended with PCBM (**a**) and bis-PCBM (**b**) after degradation under AM 1.5G or 300 lx indoor light, in air, for 60 min. EQE of the undegraded devices was used as references.

To evaluate the photoresponse of the PCDTBT:fullerene blends after degradation, the corresponding EQE curves were measured. As illustrated in Figure 4, both undegraded devices exhibit a high photoresponse in the wavelength range between 400 and 600 nm, where the peak EQE is around 550 nm with a value of ~35% for bis-PCBM cells and ~49% for PCBM cells. The shape of EQE curves did not change after degradation under 300 lx indoor light for 60 min with some decrease in intensity, while under AM 1.5G the EQE curve of the bis-PCBM device flattened in the range of 400 to 600 nm, and the EQE peak value reduced to 18 and 33% for bis-PCBM and PCBM cells, respectively. The higher EQE value of PCBM devices in each case is consistent with the *J*_SC_ results of the *J*-*V* characterizations.

### 3.3. Voltage Loss Analysis

**Figure 5:**
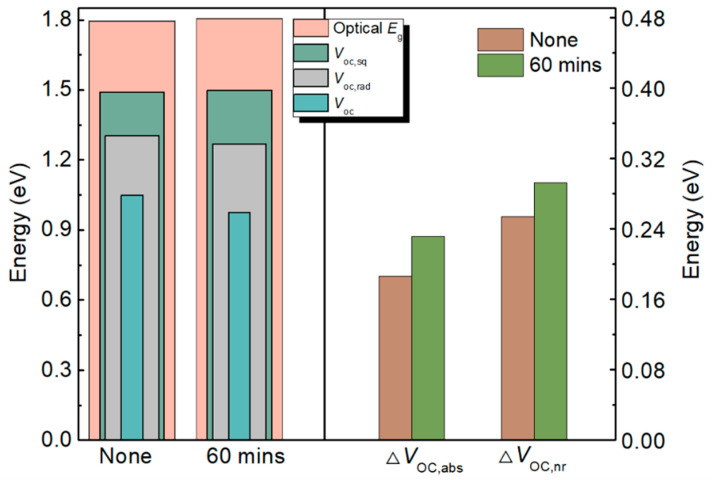
Comparison of the optical bandgap, SQ limit for *V*_OC_, radiative *V*_OC_, and *V*_OC_ for PCDTBT:bis-PCBM OPVs before and after degradation under 300 lx for 60 min. The Δ*V*_OC,abs_ = *V*_OC,sq_ − *V*_OC,rad_ and Δ*V*_OC,nr_ = *V*_OC,rad_ − *V*_OC_.

In addition to the device performance after degradation, it is also of great interest to analyze the voltage loss processes of the bis-PCBM OPV in order to understand its performance under indoor light and to maximize the *V*_OC_ by reducing the difference between the ideal and actual cases. Ideally, the OPV device can reach its maximum efficiency as indicated by the Shockley–Queisser (SQ) theory, which developed a theoretical framework to determine the limiting efficiency of a single-junction solar cell, based on the principle of detailed balance, equating the incoming and outgoing fluxes of photons for a device in open-circuit conditions [27]. For every above bandgap photon that is absorbed by the semiconductor, one electron-hole pair is generated, and all generated carriers are either collected as current in the leads or recombined, emitting a single photon per electron-hole pair. In other words, the SQ theory assumes that an ideal photovoltaic cell has a step-like absorptance with the band gap being the energy of the step, and all generated electron-hole pairs are collected with only radiative recombination occurring [28]. For the real device, it is necessary to consider the broadening of the absorption edge and the non-radiative recombination. Following previously reported methods [29,30,31], we investigated the voltage loss of PCDTBT:bis-PCBM OPVs before and after 60 min degradation under 300 lx indoor light. As illustrated in Figure 5, four loss components are summarized: the ideal maximum open-circuit voltage of SQ limit (*V*_OC,sq_), which is typically 300 mV lower than the optical band gap (*E*_g_) for the polymer:fullerene OPVs according to the previous studies in references [29]; the radiative open-circuit voltage (*V*_OC,rad_), representing the actual light absorption spectrum; the difference between *V*_OC,sq_ and *V*_OC,rad_ (Δ*V*_OC,abs_), on behalf of the absorption edge; the difference between *V*_OC,rad_ and *V*_OC_ (Δ*V*_OC,nr_), representing the non-radiative recombination. After degradation, the optical band gap *E*_g_ and *V*_OC,sq_ increased slightly due to the change of absorption, while the difference between *E*_g_ and *V*_OC,sq_ (representing the unavoidable energy loss) did not change. The *V*_OC,rad_ and *V*_OC_ decreased due to the increased loss of Δ*V*_OC,abs_ (from 0.18 to 0.23 V) and Δ*V*_OC,nr_ (from 0.25 to 0.29 V), as shown in the left part of Figure 5. The enhanced losses were due to the large shifts in energy between the main onset of absorption and the tail of absorption, and the increased non-radiative recombination after degradation. The results also indicate the increased disorders and trap states in the blend, which can slow down carrier collection and improve the likelihood of non-radiative recombination [32,33]. Reducing the intrinsic disorder of bis-PCBM and suppressing the production of new disorders during device operation can theoretically further improve the *V*_OC_ and device performance.

### 3.4. Discussion

The previous research found that the *V*_OC_ plays an important role in the OPVs under indoor light. Ensuring a high *V*_OC_ under one sun is important in achieving a high *V*_OC_ and, therefore, a high OPV device performance under low light conditions [7,34]. Bis-PCBM is a typical fullerene derivative that owns a 100 meV higher LUMO level than that of the usually used PCBM and a higher *V*_OC_ for the corresponding polymer:fullerene OPV devices under a one sun condition. Therefore, we carried out the indoor PVs study based on bis-PCBM to obtain an alternative material to PCBM and to achieve better indoor PV performance. The results show that the efficiency of the bis-PCBM devices is much worse than that of PCBM devices under strong illumination, even though the *V*_OC_ is 120 mV higher than PCBM. This is mainly due to the intrinsic disorders, low electron mobility, and amorphous property of bis-PCBM leading to adverse effects on the charge transport, charge injection and extraction, charge separation, and morphology. Under indoor light, the PCDTBT:bis-PCBM cells exhibit an expected high *V*_OC_ of up to 0.9 V under 1000 lx indoor light compared to the *V*_OC_ value of 0.79 V of PCBM-based cells. The voltage loss analysis of the bis-PCBM devices indicates a potentially higher voltage with reducing the disorders in bis-PCBM. Even though the PCE of bis-PCBM cells is still lower than that of PCBM cells (13.8 vs. 14.7%), the PCE differences between the two kinds of devices are reduced. The reason for the difference in performance between the two devices under low light illumination should be similar to that under strong illumination.

Although, at present, our bis-PCBM device does not surpass the PCBM under low light, bis-PCBM still has great application prospects in fullerene-based indoor PV. Firstly, bis-PCBM has lower electron mobility and higher molecular weight compared to PCBM, the optimal composition of bis-PCBM in the blend should be higher than PCBM, and the PCDTBT:bis-PCBM devices should have better device performance after further optimization of the fabrication process [35,36]. Secondly, bis-PCBM is a mixture of multiple isomers with energetic and structural disorders, which can be purified as isolated isomer groups or pure isomers with fewer or no disorders. The PV cells based on these isomer groups or pure isomers should have improved performance [19,37]. In terms of stability, bis-PCBM has a similar degradation rate to that of PCBM under low illumination. The top electrode and interlayer can suppress the oxygen and water permeation and decrease the degradation rate [38]. For practical application, encapsulation is also needed to protect the devices. These factors may together contribute to stable bis-PCBM OPV under an indoor environment. Moreover, the studies found that bis-PCBM is more stable than PCBM under an anaerobic environment due to the less opportunity to form dimer [26]. In addition, the OPVs may require a much shorter device lifetime considering the different target applications under indoor light (e.g., integration with consumer electronics or wireless sensors, which have a typical lifespan of less than 3–5 years) [7]. We will focus on the optimized bis-PCBM and continue to study its application in indoor OPVs in the future.

## 4. Conclusions

In summary, we carried out the performance investigation of indoor OPV devices comprised of bis-PCBM and its counterpart PCBM as the acceptor, respectively. With the advantage of high LUMO level, the PCDTBT:bis-PCBM devices yield a high *V*_OC_ up to 1.05 V and 0.9 V under AM 1.5G illumination and 1000 lx indoor light compared with the corresponding values of 0.93 V and 0.79 V of PCBM. Under AM 1.5G, the PCE of the PCDTBT:bis-PCBM cells is much lower than that of the PCDTBT:PCBM cells (2.9 vs. 4.7%), and the PCE drops faster remaining 35% of the initial value after 60 min degradation in air (remained 51% for PCBM cells). Under indoor light, the bis-PCBM and PCBM cells exhibit comparable PCE (13.8 vs. 14.7%) and device stability (maintained over 80% of the original value). The results prove that the application of bis-PCBM is an efficient and feasible approach to improve the *V*_OC_ of polymer–fullerene OPVs under both one sun and indoor light conditions. Because of the drawback of disorders and the amorphous property, the *J*_SC_ and FF of bis-PCBM devices are suppressed, leading to a lower PCE compared with PCBM devices. However, the PCE differences, as well as the stability differences between the two kinds of devices, are much reduced under indoor light. The voltage loss analysis of the bis-PCBM devices indicates a potentially higher voltage with reducing the disorders in bis-PCBM. After further optimization of the material composition and device fabrication process, bis-PCBM could be an alternative to typically used PCBM and offer great potential for high-performance OPV with enhanced *V*_OC_ and improved PCE.

## Figures and Tables

**Table 1:** The photovoltaic parameters of the OPV cells under different light sources with different light intensities. The *J*_SC_ unit mA/cm^2^ is for AM 1.5G, and μA/cm^2^ is for indoor light.

Acceptor	Light	*V*_OC_/V	FF	*J*_SC_/mA cm^−2^ μA cm^−2^ (Indoor)	PCE/%
PCBM	AM 1.5G	0.93	0.59	8.5	4.7
	300 lx	0.72	0.61	25.2	13.2
	500 lx	0.74	0.61	42.1	13.6
	1000 lx	0.79	0.62	84	14.7
Bis-PCBM	AM 1.5G	1.05	0.43	6.4	2.9
	300 lx	0.83	0.52	23.3	12
	500 lx	0.85	0.53	38.9	12.6
	1000 lx	0.90	0.55	77.7	13.8

## Data Availability

The data presented in this study are available on request from the corresponding author. The data are not publicly available due to privacy.
